# Learned Overweight Internal Model Can Be Activated to Maintain Equilibrium When Tactile Cues Are Uncertain: Evidence From Cortical and Behavioral Approaches

**DOI:** 10.3389/fnhum.2021.635611

**Published:** 2021-03-30

**Authors:** Olivia Lhomond, Benjamin Juan, Theo Fornerone, Marion Cossin, Dany Paleressompoulle, François Prince, Laurence Mouchnino

**Affiliations:** ^1^Aix-Marseille Université, CNRS, Laboratoire de Neurosciences Cognitives, FR 3C, Marseille, France; ^2^Faculty of Medicine, Department of Surgery, Université de Montréal, Montreal, QC, Canada; ^3^Institut National du Sport du Québec, Montreal, QC, Canada

**Keywords:** balance control, cutaneous plantar inputs, EEG, parietal cortex, somatosensory processes, highly trained athletes

## Abstract

Human adaptive behavior in sensorimotor control is aimed to increase the confidence in feedforward mechanisms when sensory afferents are uncertain. It is thought that these feedforward mechanisms rely on predictions from internal models. We investigate whether the brain uses an internal model of physical laws (gravitational and inertial forces) to help estimate body equilibrium when tactile inputs from the foot sole are depressed by carrying extra weight. As direct experimental evidence for such a model is limited, we used Judoka athletes thought to have built up internal models of external loads (i.e., opponent weight management) as compared with Non-Athlete participants and Dancers (highly skilled in balance control). Using electroencephalography, we first (experiment 1) tested the hypothesis that the influence of tactile inputs was amplified by descending cortical efferent signals. We compared the amplitude of P1N1 somatosensory cortical potential evoked by electrical stimulation of the foot sole in participants standing still with their eyes closed. We showed smaller P1N1 amplitudes in the Load compared to No Load conditions in both Non-Athletes and Dancers. This decrease neural response to tactile stimulation was associated with greater postural oscillations. By contrast in the Judoka’s group, the neural early response to tactile stimulation was unregulated in the Load condition. This suggests that the brain can selectively increase the functional gain of sensory inputs, during challenging equilibrium tasks when tactile inputs were mechanically depressed by wearing a weighted vest. In Judokas, the activation of regions such as the right posterior inferior parietal cortex (PPC) as early as the P1N1 is likely the source of the neural responses being maintained similar in both Load and No Load conditions. An overweight internal model stored in the right PPC known to be involved in maintaining a coherent representation of one’s body in space can optimize predictive mechanisms in situations with high balance constraints (Experiment 2). This hypothesis has been confirmed by showing that postural reaction evoked by a translation of the support surface on which participants were standing wearing extra-weight was improved in Judokas.

## Introduction

Low confidence in relying on sensory cues to control the movement prompts the nervous system to increase the weighting of predictive mechanisms. According to current models of motor control, predictions from internal models are used to avoid instabilities due to feedback delays and uncertainty (Miall and Wolpert, 1996; Franklin and Wolpert, 2011; Crevecoeur et al., 2014). For example, to distinguish linear acceleration of the body from the gravitational acceleration, Merfeld et al. (1999) showed that an internal model that mimics physical principles is used to resolve the sensory ambiguity. In addition to the sensory ambiguity problem, using prediction from internal models might also be an adaptive behavior occurring when there is low confidence on sensory cues. For example, when wearing a 20 kg vest, a depressed sensory transmission occurred (Lhomond et al., 2016) likely due to foot deformation and skin compression provoked by increased pressure on the feet (Wright et al., 2012). The perceptual consequence of the high pressure exerted on the foot sole has been described by Wu and Madigan (2014) showing that the threshold to detect force applied to the foot sole while standing increased in obese participants relative to healthy individuals.

This change (i.e., increase) in the detection threshold may be the result of a depressed signal transmission to the cortex observed by Lhomond et al. (2016) as early as 50 ms after a tactile electrical stimulation. The early cortical response was associated with the enhancement of later neural responses to tactile stimulation. However, both these early and late neural responses remain non-optimal as shown by behavioral analyses. For instance, when standing still, the postural sway was increased when healthy individuals were loaded (Lhomond et al., 2016). This is not surprising as the increased postural sway is observed in overweight individuals (Hue et al., 2007; Gravante et al., 2003; Greve et al., 2007; Teh et al., 2006; Vela et al., 1998; Wearing et al., 2006). Faster sway was also observed by Simoneau and Teasdale (2015) and was interpreted as reflecting larger balance motor command variability.

In the present study, we investigate whether the brain uses an internal model of physical laws (gravitational and inertial forces) to help estimate body equilibrium when sensory feedback from the foot tactile receptors are altered by extra-weight. The efficacy of the prediction/estimation relies on the ability to activate accurate internal representation of the body position (and motion) relative to the environment. The difference between the brain’s prediction of the consequence of the body motion and the actual motion constitutes an error signal; these sensory prediction errors drive the brain to adapt the internal model of how the loaded body motion is related to the sensory consequences (Wolpert et al., 1998a, b for review). Tactile perception may have a special role in body representation (Haggard et al., 2003), because the skin is at the interface between the body (i.e., the feet) and the outside world (i.e., supporting surface). Based on these studies, we sought to determine if the neural and behavioral responses to tactile stimulation are altered by the presence of an accurate internal model built up by training.

As direct experimental evidence for internal models is limited, we used Judoka athletes thought to have constructed internal models of external loads (i.e., opponent weight management). Therefore, it could be possible that the training of the Judokas based on the weight management of the opponent improves their ability to predict the motor and sensory consequences of a destabilization caused by an additional inertial load (Gandolfo et al., 1996; Kawato, 1999; Seidler et al., 2004). For instance, the movement of additional loads on the body is either voluntarily generated by the judoka or in reaction to the opponent’s action. Therefore, most of the training contains carrying loads (opponent) in dynamic conditions and controlling forces applied in directions other than the vertical.

Using electroencephalography (experiment 1), we tested the hypothesis that the influence of tactile inputs was amplified by descending cortical efferent signals, by measuring the amplitude of the cortical response to the electric stimulation of the plantar sole (i.e., somatosensory-evoked potential (SEP) technique). We reasoned that the amplitude of this response should be a key variable for comparing the weighting of the foot cutaneous inputs between Judokas and Non-Athlete individuals. However, to disentangle changes in processing due to a specific overweight body internal model built up by Judokas’ training or due to high-skill activity in balance control involving somatosensory afferents, we will use a “sham” group of Dancers trained in contemporary dance technique (i.e., barefoot training as in Judokas’ training). We reasoned that in Judokas the tactile-related SEP should be enhanced by cortical mechanisms aiming to compensate the depressed signal transmission due to the added weight (Lhomond et al., 2016). Alternatively, in Non-Athlete participants and Dancers the SEP should decrease due to the absence of cortical influence, that is, in absence of training in weight management. The Judokas’ training would build up an overweight-body internal model that should optimize the predictive mechanisms when loaded as compared to Non-Athlete participants. We focused our analyses on the right temporoparietal region (i.e., rTPJ), a pivotal region for processing body motion relative to the gravitational field (see Pfeiffer et al., 2014, for a review). This region showed an important role for processing information from different sensory modalities into an accurate perception of upright (Kheradmand et al., 2015). We predict that the rTPJ will show greater activity in Judokas with the added weight during natural standing.

According to current conceptions of motor control, the brain uses internal models to capture the relationship between the context in which the movement is produced and the effect of the motor commands on the moving body (Wolpert et al., 1995; Wolpert and Flanagan, 2001). The use of internal models for voluntary movement has been also extended to reflex control and specifically to the long latency reflexes of about 45-100 ms postperturbation (Kurtzer et al., 2008). Therefore, these models should also contribute to adapting postural reactions to a body movement not voluntarily produced (i.e., evoked by a translation of the support, Experiment 2). One of the goals of this study was to determine whether altered postural reaction in response to a perturbation can be explained by internal models built up during the judokas’ specific training as compared to Non-athlete participants. Postural reaction evoked by a translation of the support surface on which participants are standing wearing extra-weight should be improved in judokas.

## Materials and Methods

### Experiment 1

#### Participants and Tasks

**FIGURE 1:**
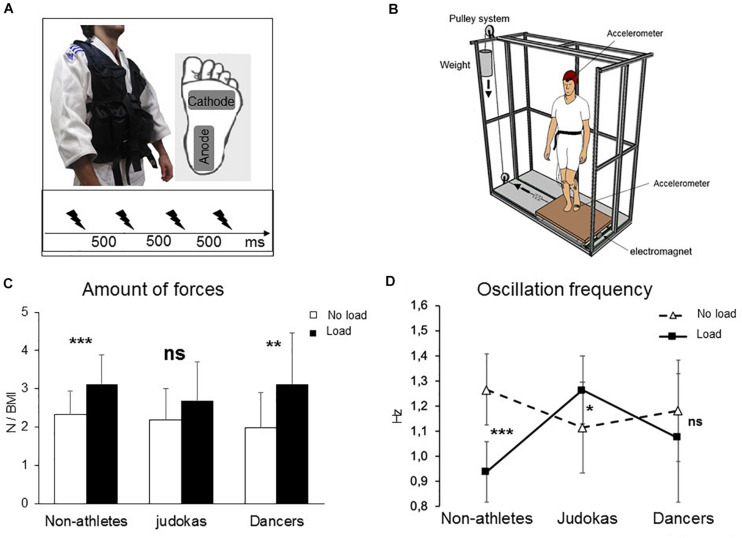
**(A)** Experimental set up for the electroencephalographic experiment. Insert depicts the workout vest worn by the participants (Judokas, Non-Athletes and Dancers); the added weight was distributed on the front and back of this vest. Position of the stimulation electrodes underneath the left foot and time-intervals between stimulations. **(B)** Experimental set up for the behavioral perturbation experiment 2. The platform translated to the right participant side by means of a pulley system. **(C)** Mean integral of the forces in all directions normalized relative to the BMI for Non-Athletes, Judokas and Dancers (error bars are standard deviation across participants) (**p < 0.005; ***p < 0.001 and ns: not significant). **(D)** Oscillation frequencies for Non-Athletes, Judokas and Dancers (error bars are standard error of the mean).

In Experiment 1, 14 highly trained male Judokas (black belt grade, mean age: 20 ± 3 years; mean height: 178 ± 10 cm; mean weight: 74 ± 10 kg) and 14 Non-Athlete participants (mean age: 25 ± 4 years; mean height: 172 ± 7 cm; mean weight: 70 ± 10 kg) participated in the experiment. A “sham” group of 5 highly trained Dancers (mean age: 25 ± 6 years; mean height: 171 ± 0.1 cm; mean weight: 58 ± 14 kg) was also tested. All participants gave their written informed consent to take part in this study, which conforms to the standards set in the Declaration of Helsinki. Participants were requested to stand upright, barefoot with their arms alongside their body and to keep their eyes closed in two conditions: i) Load, participants were standing while wearing a 20 kg weighted vest representing an increased weight of 26 ± 4% for the Judokas and 27 ± 4% for the Non-Athletes (Figure 1A) ii) No Load, without extra weight. As a result, the mean body mass index (BMI) increased from healthy weight to mild obesity for both groups (from 23.7 ± 2.3 kg/m^2^ and 23.8 ± 2.8 kg/m^2^ to 29.9 ± 2.2 kg/m^2^ and 30.1 ± 2.9 kg/m^2^ for the Judokas and Non-Athletes, respectively). For both conditions, particular attention was paid to maintain the self-selected foot position constant (i.e., feet shoulder-width apart before each trial) by marking the feet position on the platform on which they are standing. Each participant performed 15 trials in each condition for a total of 60 stimulations. The conditions were presented in block; half of the participants performed the Load condition first while the other half started with the No Load condition.

#### Stimulating System

The plantar sole of the left foot was stimulated four times (i.e., St1, St2, St3 and St4) with 500 ms between each stimulation in a recording trial which lasted 5 seconds. The stimulus was delivered by an isolated bipolar constant current stimulator (DS5 Digitimer, Welwyn Garden City, UK); the cathode was located under the metatarsal region and the anode underneath the heel (5 × 9 cm electrodes, Fyzea Optimum Electrodes). The stimulation consisted of a single rectangular 10 ms pulse. We used the technique of Mouchnino et al. (2015) who showed that stimulation of the plantar sole skin above the perceptual threshold and below the motor threshold, stimulates the plantar sole as a whole rather than targeting a specific portion of the foot. The stimulation intensity was determined as follows: for each participant while in a quiet standing position, we first found the lowest intensity which resulted in a constant perception of the stimulation. This perceptual threshold was determined, and the stimulation intensity was set at 25% higher than the threshold value. The threshold value showed a main group effect (F_2_,_30_ = 5.93; p = 0.006); *post hoc* analyses showed that the perceptual threshold of both Judokas and Dancers was higher as compared to Non-Athletes. The high perceptual threshold, which is similar (p = 0.62) for the two high-skilled populations may be the consequence of the greater thickness of the foot sole likely due to barefoot training (8.6 ± 1.3 mA, 8 ± 1.9 mA and 6.9 ± 1 mA for the Judokas, Dancers and Non-Athletes, respectively).

#### Electrophysiological Recordings and Analyses

**FIGURE 2:**
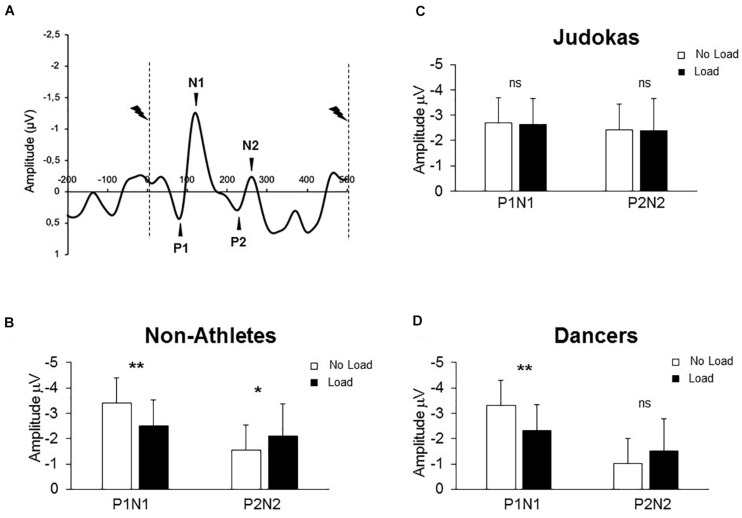
**(A)** Grand average SEP for all Non-Athletes recorded at Cz in both conditions (Control and Loaded). Dashed lines indicate the moment of the stimulation. **(B)** (Non-Athletes) **(C)** (Judokas) and **(D)** (Dancers): Mean for the 60 stimulations of the early and late SEPs amplitudes for each group (error bars are standard deviation across participants) (**p < 0.005; *p < 0.05 and ns: not significant).

Participants were fitted with a 64 Ag/AgCl surface electrodes embedded on an elastic cap ActiveTwo system, BioSemi, Netherlands or Geodesic 64-channel EEG sensor net (GSN64; Electrical Geodesics Inc., Eugene, OR, United States). The EEG was sampled at a rate of 1000 Hz. We performed data pre-processing with BrainVision Analyzer 2 (Brain Products, Germany). The EEG signals were filtered off-line with i) a 40 Hz 24 dB/octave high cut-off filters. ii) a 0.1 Hz 12 dB/octave low cut-off filters and iii) a 50 Hz notch filter when necessary. Somatosensory evoked potentials (SEPs) were obtained by averaging, for each participant and condition, all synchronized epochs relative to the electrical stimulus. The average amplitude of the 150 ms pre-stimulus epoch served as baseline. We measured the SEPs over the Cz electrode as this electrode overlays the sensorimotor cortices and on the homunculus, the feet are located on the inner surface of the longitudinal fissure. A small positive component (P1) was followed by a prominent negative deflection (N1) (Figure 2A). Thereafter, a late positive component (P2) followed by negativity (N2). The early P1N1 peak latencies were comparable to latencies evoked by stimulating the sural nerve (Altenmüller et al., 1995; Duysens et al., 1995). The fact that the sural nerve is predominantly a cutaneous nerve (Burke et al., 1981) suggests that P1N1 originates mainly from cutaneous input. The amplitude of the early P1N1 and late P2N2 SEP were measured peak-to-peak.

To estimate the neural sources of the SEPs, we used dynamic statistical parametric mapping (dSPM) implemented in the Brainstorm software (Tadel et al., 2011, freely available at http://neuroimage.usc.edu/brainstorm). We used the data from all sensors processed and averaged for each participant, condition and electrode. The forward model was computed using a three-shell sphere boundary element model (BEM) on the anatomical MRI brain MNI Colin27 template (15000 vertices), a predominant volume conductor model (Mosher et al., 1999; Huang et al., 2016). The cortical sources were searched at both the early and late components for each condition.

#### Behavioural Recordings and Analyses

Participants stood, on a 46.4 × 50.8 cm force platform (AMTI OR6-6, Watertown, MA, United States). Ground reaction forces and moments were recorded at a sampling rate of 1000 Hz and used to analyse body sway along the anteroposterior (AP) and mediolateral (ML) directions. After applying a 10 Hz 4th order Butterworth filter, data were normalized relative to the BMI of each participant. Computing the integral of horizontal forces overtime provides a mean to assess balance control as the shear forces were a technique to maintain balance (King and Zatsiorsky, 2002). For each trial, the integrals for forward, backward, rightward and leftward horizontal forces were computed after de-biasing the signal with a baseline computed during 1s from the recording onset. We computed the integral of a 2s time-window which encompassed the four stimulation periods.

### Experiment 2

#### Participants and Tasks

To assess the behavioral consequence of the neural mechanisms reported in the first experiment, we perturbed the equilibrium of standing participants while standing still. A total of 13 healthy Judokas (mean age: 22 ± 9 years; mean height: 175 ± 7 cm; mean weight: 70 ± 8 kg) and 14 healthy participants (mean age: 23 ± 3 years; mean height: 173 ± 7 cm; mean weight: 68 ± 14 kg) participate in the second experiment. No difference was found between group’s BMI (t_26_ = 0.04; p = 0.96). Seven Judokas and 1 Non-Athlete participated in both experiments on separate occasions. The participants were requested to stand upright barefoot with their arms alongside their body and to keep their eyes closed during the trial. Each participant performed 40 trials per conditions for a total of 80 trials.

#### Stimulating Systems and Behavioural Recordings and Analyses

Body translation was produced by placing the force platform on two aluminum guiding rails (Bosh Rexroth) with a ball bearing system to reduce friction. A cable attached to the platform ran laterally through a pulley system with a 1.5 kg load fixed to its extremity (Figure 1B). The platform was held stationary by an electromagnet fixed on the opposite side to the cable attachment. The platform accelerated very slightly to the right without endangering the participants’ equilibrium under the influence of the load only when the restraining electromagnet was deactivated. The lateral translation used in the current study generated a peak acceleration of 7.3 ± 9 cm.s^–2^ with the latency of 142 ± 56 ms which was well below that reported evoking a proximal hip postural reaction (13.5 cm.s^–2^, Henry et al., 1998). The average acceleration of the platform translation lasted 725 ms and did not vary from trial-to-trial within participants.

Head acceleration was recorded by using a triaxial accelerometer (Model 4630: Measurement Specialties, United States) placed on the forehead. The head acceleration and its latency were analysed in the ML direction (i.e., direction of the platform displacement). For each trial, acceleration signals were filtered with a 10 Hz 4th order Butterworth filter, de-biased and normalized relative to the BMI of each participant (i.e., including participants’ weight and height). The integrals for rightward and leftward acceleration were calculated during a 5s period. This was done to assure that at onset of the platform translation, the raw head acceleration signal was below the vestibular threshold (0.048 m.s^–2^) as reported by Gianna et al. (1996) for similar acceleration profile, so that the translation resulted in a change in the somatosensory cues from the soles of the feet without modifying vestibular inputs. Afterwards, the lateral body tilt that followed stimulated vestibular receptors as confirmed by head acceleration and most likely proprioceptive afferents.

### Statistical Analyses

In experiment 1, the SEPs and behavioral data were submitted to repeated-measures analysis of variance (ANOVA) designed with conditions (No Load and Load) and SEP component (early and late responses to tactile stimulation) and Judokas, Non-Athletes and Dancers as a group factor. We also conducted paired t-test for the statistical source estimation maps for contrasts (i.e., Loaded minus No Load conditions). In experiment 2, the data were submitted to an ANOVA design with conditions with the Judokas and Non-Athletes as a group factor. For experiments 1 and 2, significant effects were further analyzed with HSD Tukey test *post hoc* analysis. Since the sample size of the Dancer group was small, partial Eta-Squared values for the effect size were given for the ANOVA. Eta-Squared (η_P_^2^) value of 0.52 can be interpreted as 52 percent of variance that is associated with each of the main effect (Lakens, 2013). The level of significance was set at 5% for all analyses.

## Results Experiment 1

### Behavioral Measures of Postural Oscillations

To assess postural stability, the average of the integral of ML and AP forces overtime was computed and normalized to the BMI (Figure 1C). The ANOVA revealed a main condition effect (Load, No Load) (F_1_,_28_ = 30.7, p < 0.001, η_P_^2^ = 0.52). *Post hoc* analyses showed that in the Non-Athletes’ group, the amount of forces was higher in the Load than in the No Load conditions (p = 0.004) as for the Dancers’ group (p = 0.018). Interestingly, the Judokas were impervious to influence from the additional weight (p = 0.22).

Examination of the frequency of force oscillations (Figure 1D) reveals an interaction between the group and condition (F_1_,_28_ = 32; p < 0.0001, η_P_^2^ = 0.69). In Non-Athletes, *post hoc* analysis revealed a significant decrease in the Load condition compared to the No Load condition (p = 0.0001) likely due to additional inertia that tends to slow down the body oscillations. On the contrary, in Judokas, a significant increase of the frequency was observed compared to the No Load condition (p = 0.022); this likely allowed a rapid correction to the postural sway excursion due to the additional weight. No significant difference was observed in the Dancers’ group (p = 0.61).

### Somatosensory Evoked Potentials

Figure 2A shows typical EEG signals recorded during quiet standing in the Non-Athletes’ group. The results showed a significant interaction between components (early and late) condition (No Load, Load) and group (Non-Athletes, Judokas and Dancers) on the SEP amplitude (F_2_,_30_ = 13.8; p < 0.0001, η_P_^2^ = 0.47). *Post hoc* analyses confirmed that in the Non-Athlete group (Figure 2B) the early P1N1 SEP had a smaller amplitude in the Load compared to the No Load condition while the late P2N2 SEP showed greater amplitude in the Load condition (p < 0.0001 and p = 0.028, for early and late SEP respectively). In contrast in Judokas (Figure 2C), the early P1N1 or late P2N2 SEP is maintained at an equivalent value in both conditions (p > 0. 05). In Dancers (Figure 2D), solely the early SEP had a smaller amplitude in the Load compared to the No Load condition (p = 0.014) whereas the late SEP was unchanged (p > 0.05).

A main group effect was observed on the P1 latency (F_2_,_30_ = 3.78; p = 0.034, η_P_^2^ = 0.20); the early SEP latency was shorter in the Non-Athletes than in Judokas’ group (p = 0.033). No difference was observed between both highly trained groups (p = 0.96). The latencies did not show any effect of the condition (F_1_,_30_ = 1.11, p = 0.29, see Table 1 for all latencies). Overall these results confirm that training solely on equilibrium maintenance (i.e., Dance training) may not counteract the effect of extra-weight.

**TABLE 1:** Latencies and amplitude of the early and late SEPs (mean and standard deviation) in control as compared with Loaded condition for Non-Athletes, Judokas and Dancers (***p < 0.001, **p < 0.01; *p < 0.05).

Non-Athletes								
	No load				Load			
	P1N1		P2N2		P1N1		P2N2	
Latency	47 ± 11 ms	93 ± 15 ms	171 ± 40 ms	208 ± 40 ms	51 ± 10 ms	87 ± 18 ms	175 ± 40ms	215 ± 45ms
Amplitude	−3,39 ± 1,57 μV		−1,54 ± 0,84 μV		−2,50 ± 1,17 μV***		−2,10 ± 1,12 μV*	

**Judokas**								

	**No load**				**Load**			
	**P1N1**		**P2N2**		**P1N1**		**P2N2**	

	59 ± 10ms	95 ± 14ms	164 ± 36ms	220 ± 34ms	62 ± 13ms	99 ± 16ms	167 ± 23ms	224 ± 20ms
	−2,70 ± 1,28 μV		−2,43 ± 1,04 μV		−2,64 ± 1,65 μV		−2,4 ± 1,47 μV	

**Dancers**								

	**No load**				**Load**			
	**P1N1**		**P2N2**		**P1N1**		**P2N2**	

Latency	60 ± 17ms	102 ± 11ms	154 ± 29ms	178 ± 42ms	58 ± 14ms	96 ± 15ms	145 ± 29ms	175 ± 34ms
Amplitude	−3,91 ± 1,83 μV		−0,77 ± 0,68 μV		−2,68 ± 1,38 μV*		−1,03 ± 0,28 μV	

### Source Localisation

**FIGURE 3:**
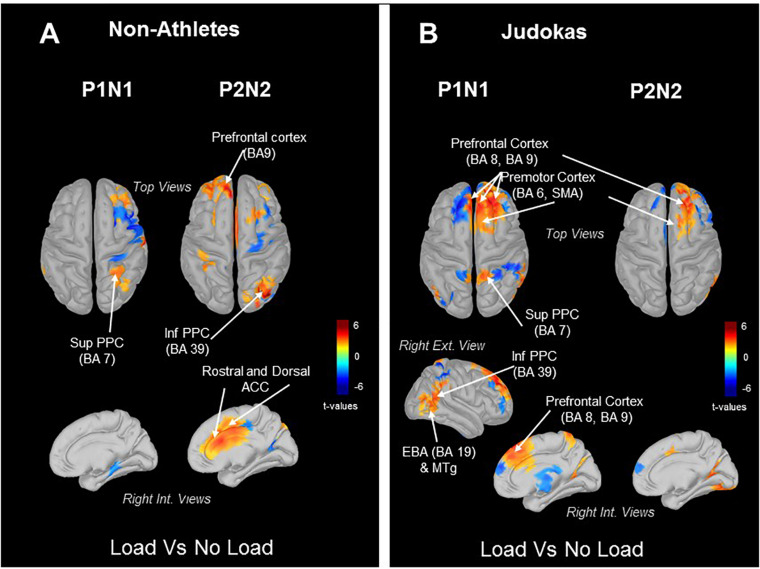
Topographic maps (dSPM) computed from all trials. Significant *t-*values (*p* ≤ 0.05, *n* = 14) of the source localization (Loaded minus Control condition) were shown at different timing to assess the dynamic of these activities during the Early SEP and Late SEP. Panel **(A)** in Judokas and panel **(B)** in Non-Athletes.

In order to highlight the sources of the early and late neural responses to tactile stimulation in the Judokas and Non-Athlete groups, we computed the statistical source maps in two 100 ms time windows encompassing the P1N1 and P2N2 components. Remarkably, in both groups (Judokas and Non-Athletes) the statistical source maps revealed as early as the P1N1 component an increase of the superior parietal cortex (BA7) in the Load relative to No Load condition (Figures 3A,B). In Judokas (Figure 3B) the increase activation of the superior PPC area mainly in the right hemisphere is associated with an increase activation of the right inferior PPC, extrastriate body area (EBA), middle temporal gyrus (MTg) and of the prefrontal and premotor areas localized bilaterally in the Load as compared to the No Load condition. Later during the P2N2 in Non-Athlete participants in the Load condition, significantly greater activation of the inferior PPC and of the prefrontal and, of the rostral and dorsal regions of the anterior cingulate cortex (ACC) bilaterally (Figure 3A; the left internal view was not shown) was observed relative to the No Load condition.

### Experiment 2: Postural Reaction Evoked by Platform Translation

**FIGURE 4:**
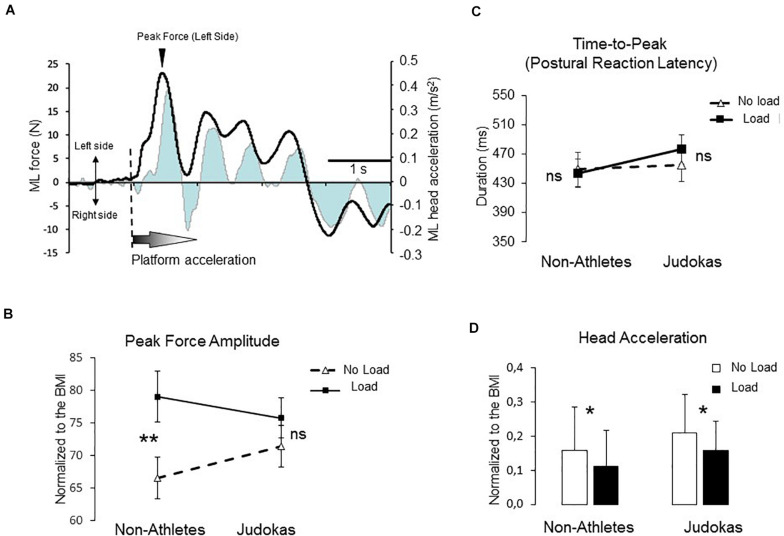
**(A)** The illustration represents the mean mediolateral force (black line) superimposed on the lateral head acceleration (gray line) for one participant. **(B)** Amplitude of the peak force for Non-Athletes and Judokas (error bars are standard error of the mean) (**p < 0.005 and ns: not significant) **(C)** Duration between the onset of the translation and the force peak (i.e., postural reaction latency) in Non-Athletes and Judokas (error bars are standard error of the mean) (ns: not significant) **(D)** Integrals of head acceleration during a 2-s period for Non-Athletes and Judokas normalized to the BMI (*p < 0.05).

When the rightward platform translation starts, the ground reaction forces increased in the opposite direction before actively reversing the forces to preserve equilibrium (i.e., postural reaction, Figure 4A). The latency of the peak of these shear forces and its amplitude were analyzed (Figures 4B,C). The time at which the forces subsequently reversed direction (i.e., postural reaction latency) was similar in both conditions (F_1_,_26_ = 0.13; p = 0.71) with no condition ^∗^ group interaction (F_1_,_26_ = 0.81; p = 0.37; overall mean 460 ± 50 ms). The ANOVA showed a main condition effect on the amplitude reached by the force (F_1_,_26_ = 15.22; p = 0.0006). However, the condition ^∗^ group interaction that failed to reach the significant level (F_1_,_26_ = 3.45; p = 0.07) showed that in the Non-Athletes’ group, the peak amplitude was higher in Load as compared to No Load condition (p = 0.002) whereas in the Judokas no differences were observed between conditions (p = 0.48).

We have been interested in the amount of head acceleration in reaction to the perturbation as an index for body sway in the frontal plane (i.e., perturbed plane, Figures 4A,D). The results showed a significant main effect of condition on the head ML acceleration (normalization with respect to the BMI) to the left side (F_1_,_26_ = 6.34 p = 0.018). The head acceleration was smaller in the Load than in the No Load conditions likely due to the stabilizing effect of the mass inertia (Figure 4D). No group effect was observed (F_1_,_26_ = 0.008; p = 0.92) on the amount of head acceleration.

## Discussion

The aim of this study was to determine if the neural and behavioral responses to tactile stimulation are altered by a specific training in weight management under high balance constraints. First, we have confirmed what has been observed previously (Lhomond et al., 2016) in Non-Athletes and extended to the Dancers. In these two groups as compared to the No Load condition, the Load condition showed a depressed early neural response to tactile stimulation. Remarkably and also in accordance to Lhomond et al.’s (2016) study, this decrease was associated with an increase in the late sensory processes (i.e., P2N2 SEP). Both mechanisms are, however, functionally non-optimal as shown by increased postural oscillations in the Load condition for Non-Athletes and Dancers. More importantly in the Judoka’s group, the neural early response to tactile stimulation was impervious to the Load condition. This suggests that the brain can selectively increase the functional gain of sensory inputs, during challenging equilibrium tasks when tactile inputs were mechanically depressed by wearing a weighted vest. From a behavioral perspective, stability was preserved in Load as compared to No Load conditions in Judokas either in conditions with a stationary supporting surface (Experiment 1) or after platform translation (Experiment 2). On the contrary, Non-Athlete participants increased the amount of force applied to the ground to stay upright (Experiment 1) and the lateral force before reversing their direction to return close to the original equilibrium position (Experiment 2).

An important result of the present study is that the early response to cutaneous stimulation (P1N1 SEP) whether it was decreased (in Non-Athletes and Dancers) or not (in Judokas) was associated with increase activation of the superior PPC (i.e., BA 7) in the Load condition. Increasing the pressure under the feet may have stimulated a population of neurons responsive to somatosensory inputs (Padberg et al., 2009) in the anterior pulvinar nucleus of the thalamus which projects to parietal areas 5 and 7 (i.e., superior PPC; Pons and Kaas, 1985; Gharbawie et al., 2010). This direct thalamocortical projection to the superior PPC could be activated when compression under the skin of the feet increases due to the loading. Indeed, the superior parietal lobule (e.g., PEc and PE areas in the macaque brain), which is an important node for sensorimotor integration, receives strong afferents from regions of the thalamus where legs are represented (Chung et al., 1986; Impieri et al., 2018). Although the same increase of activation is observed in the Load condition in both Non-Athletes and Judokas, the different responses to tactile stimulation can be explained by the presence (Judokas) or not (Non-Athletes) of a parallel distributed processing within the cerebral cortex. For instance, increase activities in the prefrontal cortex (PFC), premotor cortex, inferior PPC, EBA and MTg in the Load condition were observed in Judokas.

In Judokas, in the Load condition the PFC come into play as early as the P1N1. This is consistent with the involvement of the PFC when balance requires a high attention level in order to trigger the adapted postural reactions. For instance, Basso Moro et al. (2014) showed that the PFC increases its activity in quiet standing participants asked to maintain their ‘virtual” equilibrium viewing a virtual swing board susceptible to external destabilizing perturbations. Previously, Slobounov et al. (2000, 2005) proposed the existence of specialized neural detectors of imbalance. They reported an increase of power of the gamma band frequency (30–50 Hz) over the PFC about 200 ms before an avatar shifted from a stable to an unstable posture leading to fall. More specifically the link between PFC activation and the amplitude of the cortical response to tactile stimulation can be described as spatial attention. Indeed, spatial attention to a stimulus (here tactile) enhances the response to the stimulus as reported by Chen et al. (2008) for primary visual areas. The attentional gain operating at cortical level may have counteracted the depressed transmission of tactile inputs due to increase pressure on the mechanoreceptors embedded in the foot sole. For example, this cortical modulation has been reported to maximize the quality of the tactile feedback and improves tactile perception prior to foot contact during locomotion (Duysens et al., 1995 and Dietz et al., 1985).

In addition, there is growing evidence that the frontoparietal cortical network strongly lateralized to the right hemisphere observed in Judokas is recruited for spatial attention when information about the direction of motion is known in advance (see for review Corbetta and Shulman, 2002). Indeed expectation for the pressures motion under the feet when wearing the weighted vest has been learned during the Judokas’ training. For instance, the movement of additional loads on the body is either voluntarily generated by the judoka or in reaction to the opponent’s action. Therefore most of the training contains carrying loads (opponent) in dynamic conditions and applied in directions other than the vertical.

Expected tactile stimuli (e.g., location, features) elicit greater neural activity in several cortical areas involved in the dorsal frontoparietal network, than unexpected stimuli as in Non-Athletes in the Load condition. These results suggest that Judokas have built up an internal representation of the somatosensory disturbance due to the added weight on the body as it is evidenced by the high balance stability observed in Judokas in the Load condition (Experiment 1) and by the fact that the lateral force before triggering the postural reaction in response to the platform translation was impervious to the load condition (Experiment 2). The fact that at least two representations (normal weighted and overweighed representations) cohabit is in line with evidence that representations of the self are malleable rather than fixed (see for review Apps and Tsakiris, 2014).

Our results highlight the well-known role of the right parietal areas (inf PPC and EBA) in building up, storing and updating a body representation in space (Wolpert et al., 1998a,b; Tsakiris et al., 2008; Pfeiffer et al., 2014). For example, the disruption of the rTPJ and specifically the BA 39 area (see MNI coordinates, Tsakiris et al., 2008) suggested that the rTPJ is actively involved in maintaining a coherent sense of one’s body distinct from the external world. An efferent signal from the right PPC could have allowed the integration of tactile cues with proprioceptive localization of body parts in space leading to the remapping of tactile stimulation into an external reference frame (Azañón et al., 2010; Kammers et al., 2006). This is in line with Fabre et al.’s (2020b) study showing that obese individuals improved the sensory processes used in the setting of the anticipatory postural adjustments prior to step initiation after following a program targeting different dimensions of the body internal representation (e.g., proprioceptive training, dance and dance-like training, physical activity and mindfulness-Based Stress Reduction and relaxing session) and not weight loss.

One could argue that the early cortical response to the tactile stimulation observed in Judokas reflected the acquisition of high-level motor skill in balance control. It is noteworthy, however, that even though the Dancers (Perrin et al., 2002; see Bläsing et al., 2012 and Fabre et al., 2020a for reviews) like the Judokas (Taskin et al., 2015) were highly trained in balance control they showed similar neural and behavioral responses to the Non-Athletes’ group in the Load condition. It rules out the possibility that early neural modulation reflects solely a learned motor skill in balance control. One could argue that both behavioral and cortical response in dancers might be attributed to their small sample size and/or to the fact that the weight they carried was heavier relative to their body weight than judoka’s. However, the similarities in both behavior and cortical activities between the Non-athlete group and the dancers (corroborated by high Eta-squared values), does not support this limitation. Rather, we hypothesize that the sensorimotor changes observed in judokas and not in dancers, which enhanced relevant foot cutaneous information were linked to a modified body representation or a more efficient use of this representation.

Finally, the fact that in Judokas, premotor areas are also engaged in this frontoparietal network shows the close functional linkage between the stimulus- and response-selection signals. In support of this close connection, we have recently demonstrated (Lhomond et al., 2019) that premotor (i.e., SMA) was at the origin of the upregulating cutaneous inputs (when mechanically depressed by added weight) to restore the level of sensory afferents to make it suitable for setting the anticipatory adjustments prior to step initiation. The relationship between relevant sensory inputs and motor activity is evidenced by the fact that Judokas increased their frequency of postural oscillations in the Load as compared to the No Load condition. This enhancement of the frequency is likely actively triggered because due to the added mass the frequency should mechanically decrease as observed in Non-Athletes. In addition, this could explain that the amplitude of the destabilization (i.e., peak force, Figure 4B) following the platform translation (experiment 2) before the postural reaction is triggered, were not affected by the Load condition in Judokas. By contrast, the delayed postural responses (larger amplitudes of destabilization) in the Load condition observed in Non-Athletes may be the consequence of a compensation that comes later (i.e., P2N2). The greater activation in the Load condition during the late P2N2 component (Figure 3A) of the right inferior PPC and the dorsal and rostral cingulate cortices (ACC) of both hemispheres is consistent with the fact that the motor commands issued from the ACC may have increased due to the load management. This is possible by the existence of direct bilateral connections of this motor area of the medial wall with the spinal cord and the primary motor cortex (He et al., 1995; Dum et al., 2016).

Our study is the first to explore the cortical activity relative to a judo training which permits to develop some abilities in postural stability during a task requiring a high level of balance control in load condition. Our results showed that the Judokas use internal model of the extra weight built during their training to help estimate body equilibrium when sensory feedback from the foot tactile receptors is altered by extra loads. Judo is one of the Paralympic disciplines that include visually impaired athletes. One particular interesting application of this study is how visually impaired Judokas used the sensory feedback from their feet in order to maintain balance and execute accurate movement carrying extra weight. Considering this study as a translational research and applying knowledge for example, to military purpose when the weight the soldiers carry is heavy is, however, premature. The results of this study may be dependent on the duration of the ‘over-weighted balance training’ targeting internal body representation improvement. So far, there are no scientific guidelines concerning the optimal duration and intensity of exercises with dynamic external load.

## Data Availability Statement

The raw data supporting the conclusions of this article will be made available by the authors, without undue reservation.

## Ethics Statement

Ethical review and approval was not required for the study on human participants in accordance with the local legislation and institutional requirements. The patients/participants provided their written informed consent to participate in this study.

## Author Contributions

OL, FP, and LM contributed to the design, data collection and analyses, and writing of the manuscript. BJ, TF, MC, and DP contributed to the set up design, data collection, and results analyses. All authors contributed to the article and approved the submitted version.

## Conflict of Interest

The authors declare that the research was conducted in the absence of any commercial or financial relationships that could be construed as a potential conflict of interest.
